# Multi-epitope vaccine candidates based on mycobacterial membrane protein large (MmpL) proteins against *Mycobacterium ulcerans*

**DOI:** 10.1098/rsob.230330

**Published:** 2023-11-08

**Authors:** Tamara Z. Ishwarlall, Victoria T. Adeleke, Leah Maharaj, Moses Okpeku, Adebayo A. Adeniyi, Matthew A. Adeleke

**Affiliations:** ^1^ Discipline of Genetics, School of Life Sciences, University of KwaZulu-Natal, Durban, South Africa; ^2^ Department of Chemical Engineering, Mangosuthu University of Technology, Umlazi, Durban, South Africa; ^3^ Department of Chemistry, Faculty of Natural and Agricultural Sciences, University of the Free State, Bloemfontein, South Africa; ^4^ Department of Industrial Chemistry, Federal University Oye Ekiti, Ekiti State, Nigeria

**Keywords:** Buruli ulcer, immunoinformatics, *Mycobacterium ulcerans*, vaccine design

## Abstract

Buruli ulcer (BU) is a neglected tropical disease. It is caused by the bacterium *Mycobacterium ulcerans* and is characterized by skin lesions. Several studies were performed testing the Bacillus Calmette-Guérin (BCG) vaccine in human and animal models and *M. ulcerans-*specific vaccines in animal models. However, there are currently no clinically accepted vaccines to prevent *M. ulcerans* infection. The aim of this study was to identify T-cell and B-cell epitopes from the mycobacterial membrane protein large (MmpL) proteins of *M. ulcerans.* These epitopes were analysed for properties including antigenicity, immunogenicity, non-allergenicity, non-toxicity, population coverage and the potential to induce cytokines. The final 8 CD8^+^, 12 CD4^+^ T-cell and 5 B-cell epitopes were antigenic, non-allergenic and non-toxic. The estimated global population coverage of the CD8^+^ and CD4^+^ epitopes was 97.71%. These epitopes were used to construct five multi-epitope vaccine constructs with different adjuvants and linker combinations. The constructs underwent further structural analyses and refinement. The constructs were then docked with Toll-like receptors. Three of the successfully docked complexes were structurally analysed. Two of the docked complexes successfully underwent molecular dynamics simulations (MDS) and post-MDS analysis. The complexes generated were found to be stable. However, experimental validation of the complexes is required.

## Graphical abstract

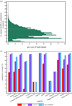

## 1. Background

Vaccination is a vital part of healthcare for its role in preventing the progression of disease in a host. Since the initial use of the term ‘vaccine’ by Edward Jenner in 1796, it has led to the eradication of smallpox, the nearly complete elimination of polio and is estimated to have reduced the cost of illnesses and hospitalizations by more than $500 billion in 94 low- and middle-income countries [1–3]. Vaccine development can be carried out through the use of various methods resulting in carbohydrate vaccines, DNA vaccines, live attenuated viral vaccines, inactivated vaccines and recombinant vaccines, among others [4]. It is important to remember that while these types of vaccines are effective, there are several infectious diseases against which there are no effective vaccines [1]. There are also several challenges regarding vaccine development. This includes the long periods needed for candidate screening, production, clinical development and safety screening [5]. This has a greater impact, especially in current times, with the faster spread and mutation of diseases resulting in a need for more rapid vaccine development. However, this is not to say that the complete fast-tracking of vaccine development is to be encouraged, as this may result in ineffective vaccines or the development of unexpected side effects [6]. Another challenge to vaccine development is the accurate reproduction of native antigens and an adequately strong immune response [5]. This, in turn, requires an in-depth understanding of the mechanisms behind a protective immune response against the target pathogen, without which there may be possible negative side effects or poor clinical results [7]. The advancement of various technologies in the immunology, microbiology, genetics and structural biology fields has led to the development of new approaches to vaccine development, such as bioinformatics, systems biology and high-throughput screening methods [7,8]. The use of such high-throughput screening technologies may accelerate the pre-clinical phase of vaccine development [9].

Various new technologies used in vaccine research include genomics, transcriptomics, proteomics, metabolomics and immunomics [10]. These ‘vaccinomics’ can be used to identify promising candidates from the target organism genome using *in silico* bioinformatics [10]. One such approach is reverse vaccinology (RV), which allows for the selection of suitable antigens prior to experimental testing [11,12]. This approach overcomes the limitations of traditional vaccines in terms of reducing antigenic variability and molecular mimicry [9]. It also allows for the development of vaccine candidates that may be effective against a range of strains of a pathogen [13]. For the development of antimicrobial vaccines, it reduces the need for bacterial culturing at the screening stage of analysis [13]. This approach may also reduce the cost of the initial stages of vaccine development [9]. However, this approach remains highly dependent on experimental studies, especially when examining the various aspects of the identified antigens, such as their functions, properties, structures, effect on virulence, and if they interact with the host [12]. This approach is attractive as an initial design step, especially for diseases for which there are no vaccines or neglected diseases.

The causative agent of the neglected tropical disease Buruli ulcer (BU) is *Mycobacterium ulcerans* [14]. Symptoms appear on the skin and subcutaneous tissue, with the formation of nodules, plaques, oedemas and skin ulcerations [15]. However, treatment may be delayed due to the often painless progression of the lesions [15,16]. This delay may result in disability surrounding joint movements or severe deformity [17]. Previous treatment consisted of the removal of the lesions through surgery; however, it has now developed primarily to the use of antibiotics, with surgery being used to remove necrotic tissue [18]. The current antibiotic regimen recommended by the World Health Organization (WHO) [14] is the use of 10 mg kg^−1^ of rifampicin once daily combined with 7.5 mg kg^−1^ clarithromycin twice daily. Early diagnosis and treatment are critical in minimizing the symptoms of this disease [18]. However, BU often occurs in resource-limited communities that often have limited access to healthcare [19]. Furthermore, the lack of complete knowledge regarding the route of transmission or vectors continues to hinder prevention and control strategies [20].

Presently, there are no clinically accepted BU vaccines available. The Bacillus Calmette-Guérin (BCG) vaccine used against tuberculosis is the only vaccine tested in humans against BU [21,22]. However, it has been concluded that current BCG vaccines cannot fully protect against BU in humans [23]. Additional studies using *M. ulcerans-*specific vaccines have been carried out in various animal models; however, long-term protection was not observed [24–36]. The RV approach has been applied in two studies at present [37,38]. These studies successfully identified T-cell and B-cell epitopes from the proteomes of *M. ulcerans.* The first study focused on chromosome-encoded virulence proteins [37], while the second study analysed proline-glutamate polymorphic GC-rich sequence (PE-PGRS) family proteins [38]. Our previous study focused on identifying epitopes from the major facilitator superfamily (MFS) transporter protein of *M. ulcerans* [39]. The identified epitopes [37] and vaccine candidates constructed by [38,39] displayed several desirable properties. However, several proteins within the *M. ulcerans* proteome remain to be studied.

One such set of proteins is the mycobacterial membrane protein large (MmpL) proteins. These transporters are a subclass of the resistance-nodulation-cell division (RND) superfamily [40]. The MmpL proteins play a critical role in the establishment of the mycobacterial cell envelope through the translocation of complex envelope lipids, which are associated with virulence, and siderophores through the plasma membrane to the periplasmic space [41]. The importance of their role highlights their potential as vaccine candidates. MmpL transporters can be identified as potential important virulence factors [40]. Identifying proteins and resulting antigens with an outer membrane topology is advantageous, as these are more accessible to the host's immune system [37]. The aim of this study is to identify CD8^+^ and CD4^+^ T-cell and B-cell epitopes with desirable properties from the MmpL proteins of *M. ulcerans*. Once screened, these epitopes are used to construct vaccine candidate sequences and docked with Toll-like receptors (TLRs). These constructs and complexes undergo analysis to determine their capability to produce a protective immune response against *M. ulcerans* infection.

## 2. Methods

### 2.1. T-cell and B-cell epitope analysis

#### 2.1.1. Identification of potentially virulent outer membrane peptides

Twelve *M. ulcerans* genomes were extracted from the National Center for Biotechnology Information database (NCBI) (https://www.ncbi.nlm.nih.gov/) (electronic supplementary material, table S1). They were downloaded in protein format and submitted to the MP3: Prediction of Pathogenic/Virulent Proteins database (http://metagenomics.iiserb.ac.in/mp3/index.php), with the threshold set to 0.5 [42]. Potentially pathogenic proteins are predicted using an integrated support vector machine (SVM)–hidden Markov model (HMM) approach [42]. Only MmpL proteins that were identified as potentially virulent were selected. These proteins were submitted to Clustal Omega v 1.2.4 (https://www.ebi.ac.uk/Tools/msa/clustalo/) [43]. Once aligned, the proteins were analysed using the TMHMM v 2.0 webserver (https://services.healthtech.dtu.dk/service.php?TMHMM-2.0) [44,45] to identify proteins that have an outer topology.

#### 2.1.2. Identification of human leucocyte antigen alleles for T-cell epitope prediction

In order to determine the human leucocyte antigen (HLA) alleles to be selected for analysis, the Allele Frequency Net Database (http://www.allelefrequencies.net/default.asp) [46] was used to identify HLA alleles within endemic countries. These alleles were filtered based on the allele frequency, in order of highest to lowest, and the population standard was set to gold only (electronic supplementary material, table S2). The top 10 HLA allele types for each endemic country were selected and used to generate the respective CD8^+^ and CD4^+^ epitope peptides, based on the availability of the HLA alleles on the respective websites (electronic supplementary material, table S3) [46–48].

#### 2.1.3. Prediction of T-cell epitopes

##### 2.1.3.1. Prediction of CD8^+^ T-cell epitopes

The identified potential outer membrane proteins were extracted and submitted to the NetMHCpan v 4.1 website (https://services.healthtech.dtu.dk/service.php?NetMHCpan-4.1), with the peptide length set to 9 [47]. Using the chosen HLA alleles (electronic supplementary material, table S3), peptides capable of binding to MHC I were generated. Only peptides with IC_50_ values ≤ 250 nM were extracted and submitted to VaxiJen v 2.0 (http://www.ddg-pharmfac.net/vaxijen/VaxiJen/VaxiJen.html) for antigenicity analysis [49]. The target organism was set to bacteria, with a threshold of 0.5. Peptides with an antigenicity value ≥ 0.5 were extracted. The antigenic nanomers were then analysed using the Class I Immunogenicity Web server (http://tools.iedb.org/immunogenicity/) [50], following which peptides with positive scores were selected.

##### 2.1.3.2. Prediction of CD4^+^ T-cell epitopes

The identified potential outer membrane proteins were also submitted to NetMHCIIpan v 4.0 (https://services.healthtech.dtu.dk/service.php?NetMHCIIpan-4.0) to predict which peptides would be capable of binding to MHC II molecules of known sequences [48]. The identified HLA alleles were selected (electronic supplementary material, table S3) and the peptide length was set to 15. Upon completion of the analysis, only peptides with IC_50_ values ≤ 250 nM were extracted. These peptides underwent antigenicity analysis using VaxiJen v 2.0 (http://www.ddg-pharmfac.net/vaxijen/VaxiJen/VaxiJen.html) [49]. The target organism selected was bacteria, and the threshold was set to 0.5. Only antigenic epitopes (≥ 0.5) were selected.

#### 2.1.4. Assessment of cytokine-induction, allergenicity, toxicity and conservancy properties of the CD4^+^ T-cell epitopes

The identified antigenic CD4^+^ epitopes were submitted to the IFNepitope Web server (http://crdd.osdd.net/raghava/ifnepitope/predict.php) [51]. Epitopes identified as being capable of inducing interferon-gamma (IFN-γ) were selected. These epitopes were submitted to IL4pred (https://webs.iiitd.edu.in/raghava/il4pred/predict.php) [52]. The criteria were kept at default. Only epitopes predicted to be capable of inducing interleukin 4 (IL-4) were extracted from the results. The identified immunogenic CD8^+^ and cytokine-inducing CD4^+^ epitopes were submitted to AllerTOP v. 2.0 Web server (https://www.ddg-pharmfac.net/AllerTOP/) [53]. This was done to analyse the potential allergenic or non-allergenic nature of the peptides. Only non-allergenic epitopes were selected and submitted to ToxinPred (https://webs.iiitd.edu.in/raghava/toxinpred/index.html) [54,55] for toxicity analysis. Non-toxin epitopes were selected and submitted for conservancy analysis using the Epitope Conservancy Analysis Web server (http://tools.iedb.org/conservancy/) [56]. CD8^+^ epitopes with 100% linear conservancy were identified and used to identify overlapping CD4^+^ epitopes. Due to the large quantity of T-cell epitopes, conserved epitopes with an antigenicity score ≥ 1.5 were selected. Conservancy analysis (http://tools.iedb.org/conservancy/) and identification of CD4^+^ T-cell epitopes were performed on the filtered epitopes [56]. The source proteins of the final T-cell epitopes were identified from the potentially virulent MmpL proteins.

#### 2.1.5. Population coverage analysis

The conserved T-cell epitopes were submitted to the Population Coverage Web server (http://tools.iedb.org/population/) [57]. The 8 CD8^+^ and 12 CD4^+^ T-cell epitopes and their respective HLA alleles were inputted (electronic supplementary material, table S4). The endemic regions were chosen per the WHO database [58]. The following regions were selected: World, Australia, Cameroon, Central African Republic, Congo, Côte d'Ivoire, Ghana, Gabon, Japan, Liberia, Nigeria, Papua New Guinea, Sudan and West Africa. The population coverages for class I, class II and class combined were estimated.

#### 2.1.6. Prediction of B-cell epitopes

The initially identified outer membrane peptides were submitted to ABCpred (https://webs.iiitd.edu.in/raghava/abcpred/index.html) to identify potential B-cell epitopes [59,60]. The analysis parameters were kept at the default, and only linear B-cell epitopes with a score ≥ 0.51 were extracted. These B-cell epitopes were submitted to VaxiJen v 2.0 (http://www.ddg-pharmfac.net/vaxijen/VaxiJen/VaxiJen.html) [49] for antigenicity analysis. The previous parameters were maintained. B-cell epitopes with an antigenicity score ≥ 0.50 were selected. These epitopes were submitted to the Epitope Conservancy Analysis Web server (http://tools.iedb.org/conservancy/) [56] along with the previously identified conserved 8 CD8^+^ epitopes. CD8^+^ epitopes with a conservancy of 100% were selected and used to identify overlapping B-cell epitopes. The conserved B-cell epitopes were submitted to AllerTOP (https://www.ddg-pharmfac.net/AllerTOP/) for allergenicity analysis and ToxinPred (https://webs.iiitd.edu.in/raghava/toxinpred/index.html) for toxicity analysis [53–55]. A total of 5 non-allergenic and non-toxic conserved B-cell epitopes were identified from the analysis. These epitopes were used to identify the potentially pathogenic source proteins.

#### 2.1.7. Molecular docking of the CD8^+^ and CD4^+^ T-cell epitopes

The molecular docking of the conserved T-cell epitopes to their respective HLA alleles was performed to observe possible interactions between the epitopes and the respective MHCs [61]. The most conserved HLA alleles for the CD8^+^ T-cell epitopes were HLA-A*23:01, HLA-A*24:02, HLA-C*24:06 and HLA-C*24:13. HLA-A*24:02 was chosen, and the corresponding crystalline structure 5WWJ [62] was downloaded from the RCSB Protein Data Bank (PDB) (https://www.rcsb.org/) [63,64]. The most conserved HLA allele for the CD4^+^ T-cell epitopes was HLA-DRB1*15:01. The crystalline structure 5V4M [65,66] was also downloaded from the RCSB PDB (https://www.rcsb.org/) [63,64]. Both structures were analysed and prepared for docking using UCSF Chimera v.1.14 [67]. Preparation consisted of binding analysis and docking prep on chain A for HLA-A*24:02 and chains A and C for HLA-DRB1*15:01. The NACCESS 2.1.1 package [68] was used to determine the solvent accessibility and flexibility of the binding sites. Zone analysis was performed on chains E and C of HLA-A*24:02 and HLA-DRB1*15:01, respectively. The crystalline structure and the predicted T-cell epitopes were submitted to ATTRACT Online (http://www.attract.ph.tum.de/services/ATTRACT/peptide.html) for docking analysis. The docking was completed on the locally installed ATTRACT on the Centre for High-Performance Computing (CHPC) South Africa. Upon completion of docking, there were 50 frames generated for each docking interaction for the T-cell epitopes. The frame with the lowest binding energy was selected for the CD8^+^ and CD4^+^ T-cell epitopes. Visual Molecular Dynamics (VMD) software v 1.9.3 was used to visualize the best model for each epitope, and UCSF Chimera v.1.14 was used to produce images of the epitope structures [67,69]. LigPlot^+^ v. 2.2.5 was used to generate a ligplot for the CD8^+^ T-cell epitope with the lowest binding energy [70]. The BIOVIA Discovery Studio Visualizer 2021 v 21.1.0.20298 was used to visualize the hydrogen bonds between the CD4^+^ T-cell epitope with the lowest binding energy and the respective HLA allele [71].

### 2.2. Construction of multi-epitope vaccine candidate sequences and structural analysis

#### 2.2.1. Generation of the multi-epitope vaccine sequences

The final number of epitopes consisted of 8 CD8^+^ and 12 CD4^+^ T-cell and 5 B-cell epitopes. These epitopes were used to construct 5 candidate vaccine sequences. The adjuvants chosen consisted of human β-defensin 2 (hBD2), and the TLR 2 and 4 agonists lipoprotein LprG [72] and the resuscitation-promoting factor (RpfE), respectively [73]. These adjuvants were chosen to boost the potential immune response. The sequences for hBD2 (accession number AAN64161.1), LprG (accession number ABL04283.1) and RpfE (accession number OIN23277.1) were retrieved from NCBI (https://www.ncbi.nlm.nih.gov/). Various linkers were used between the adjuvant, CD8^+^, CD4^+^ T-cell epitopes and the B-cell epitopes (electronic supplementary material, table S5). The epitopes were ordered per decreasing antigenicity value. The 5 models were submitted to AllerTOP v. 2.0 (https://www.ddg-pharmfac.net/AllerTOP/) [53] and VaxiJen v 2.0 (http://www.ddg-pharmfac.net/vaxijen/VaxiJen/VaxiJen.html) [49], with the previously stated parameters.

#### 2.2.2. Structural analysis of the vaccine candidate sequences

The constructs were submitted to the Gor IV Secondary Structure Prediction Method Web server (https://npsa-prabi.ibcp.fr/cgi-bin/npsa_automat.pl?page=/NPSA/npsa_gor4.html) to predict the secondary structures [74,75]. The vaccine constructs were submitted to GalaxyTBM on the GalaxyWeb Web server (https://galaxy.seoklab.org/cgi-bin/submit.cgi?type=TBM) [76–80] for structure prediction and initial refinement. The best models for each construct were selected and submitted to GalaxyRefine (https://galaxy.seoklab.org/cgi-bin/submit.cgi?type=REFINE) [81,82] for further refinement. Models were chosen based on their conformation and submitted to ProSA-web (https://prosa.services.came.sbg.ac.at/prosa.php) for validation [83,84]. Further validation was carried out using ERRAT and PROCHECK on the SAVES v6.0 Web server (https://saves.mbi.ucla.edu/) [85–87].

#### 2.2.3. Physico-chemical analysis of the vaccine candidate sequences

The candidate sequences were submitted to the ProtParam tool (https://web.expasy.org/protparam/) [88] for analysis of their *in silico* physical and chemical properties. These properties consist of molecular weight, amino acid composition, isoelectric point, instability index, aliphatic index, the grand average of hydropathicity (GRAVY) and the estimated half-life of the protein in mammalian cells, yeast cells, and *Escherichia coli* [88]. The models were then analysed using the SCooP v 1.0 website (http://babylone.ulb.ac.be/SCooP/index.php) [89–91]. Various thermodynamic properties of the structures, such as the melting temperature (*T*_m_), change in enthalpy (*Δ*H_m_), change in specific heat upon folding (*Δ*C_p_), and the folding free energy at room temperature (*ΔG*_r_), are determined by this website [89–91].

#### 2.2.4. *In silico* codon adaptation, vaccine optimization and expression

The JAVA Codon Adaption Tool (JCat) (http://www.jcat.de/) [92] was used to perform codon adaptation and vaccine optimization. *Escherichia coli* (strain K12) was selected as the model organism. This organism was selected due to the dissimilarity between its codon usage and humans, which may result in higher expression [93]. The restriction sites HindIII (5'AAGCTT'3) and BamHI (5'GGATCC'3) were added to the N- and C-terminals of all the optimized DNA sequences, respectively. The insertion of the candidate sequences into the pET-28a(+) vector was carried out using SnapGene v.6.0.2 software (from Insightful Science; available at https://www.snapgene.com/).

#### 2.2.5. Molecular docking of the multi-epitope sequences to toll-like receptors

The crystalline structures for TLR2 (3A7B) [94,95] and TLR4 (4G8A) [96,97] were downloaded from RCSB PDB (https://www.rcsb.org/), respectively [63,64]. UCSF Chimera v.1.14 was used to analyse the structures and perform docking prep on chain A for both structures [67]. The structures were submitted to the CHPC for analysis using the NACCESS 2.1.1 package [68]. However, NACCESS analysis was unsuccessful for constructs 3 and 4 due to the max cubes being exceeded. The docking method followed that of the T-cell epitopes for constructs 1, 2 and 5. Construct 1 was docked with TLR2, while constructs 2 and 5 were docked with TLR4.

#### 2.2.6. Structural analyses of the TLR–multi-epitope vaccine complexes

The CABSflex v. 2.0 website (http://biocomp.chem.uw.edu.pl/CABSflex2) [98] was used to analyse the flexibility of the three multi-epitope vaccine (MEV)–TLR complexes. The binding interactions within the complexes were analysed using ProFunc (https://www.ebi.ac.uk/thornton-srv/databases/profunc/) [99] and viewed using PDBsum [100]. AGGRESCAN3D v. 2.0 (http://212.87.3.12/A3D2/) [101] was used to determine the solubility and aggression propensity of the complex.

#### 2.2.7. Immune simulations

C-IMMSIM (https://kraken.iac.rm.cnr.it/C-IMMSIM/index.php?page=1) was used to simulate potential immune responses to MEV1, MEV2 and MEV5 [102,103]. The parameters were left as the default settings, with a time step of 1 and a single injection with no lipopolysaccharide (LPS).

### 2.3. Molecular dynamics simulations

Molecular dynamics simulations (MDS) using the AMBER 14 and 18 packages were performed on the docked complexes, bound, and unbound MEV constructs [104,105]. MDS are used to determine the stability of the complexes and the interactions between the proteins [106]. The proteins were described using FF14SB [107]. The topologies were generated using the LEaP module of AMBER 14 [104]. Protons and Na^+^ ions were added as counter ions to the complexes, and Cl^−^ was added to the unbound MEV constructs. This was done to neutralize the system in an orthorhombic box of TIP3P water molecules of 8 Å [108]. Initial energy minimization was carried out for 10 000 steps (500 steepest descents with 9500 conjugate gradient). However, initial energy minimization failed to run for construct 2. Full energy minimization was carried out for 2000 steps for constructs 1 and 5. The complexes were gradually heated from 0 K to 300 K in a canonical ensemble (NVT) with a Langevin thermostat for 2 ns. The collision frequency applied to the system was 1.0 ps^−1^, with the density of the water system regulated with 2 ns of NPT (constant number N, pressure P and time T) simulation. The complexes were equilibrated at 300 K for an additional 2 ns at a pressure of 1 bar. The production stage was run for 100 ns at NVT. The simulations were run using the GPU (CUDA) version of PMEMD provided in AMBER 18 [105,109–111].

### 2.4. Post-molecular dynamics simulations analysis

Post-MDS analysis was performed using the CPPTRAJ and PTRAJ modules in AMBER 18 [105,112]. The root mean square deviation (RMSD) and the root mean square fluctuations (RMSF) of the complexes and the MEV constructs were determined. The Molecular Mechanics/Generalized Born Surface Area (MM/GBSA) module in AMBER 18 was used to calculate the endpoint binding free energy of the docked complexes using the formula

$$\Delta G_{\mathrm{bind}}=G_{\mathrm{complex}}-(G_{\mathrm{receptor}}+G_{\mathrm{ligand}}).$$

The CPPTRAJ and PTRAJ modules generated 2000 frames of the complexes and the unbound MEVs [112]. VMD v 1.9.3 was used to view the generated structure [69]. The Bio3D package was loaded onto RStudio v 4.0.4 and used to perform principal component analysis (PCA) and cross-correlation analysis [113,114]. PCA provides insight into the nature of the clusters and conformational variations that occurred after MDS [115]. Through the analysis of pairwise cross-correlation coefficients, cross-correlation analysis generates a dynamical cross-correlation matrix (DCCM) [113]. This can be used to determine the magnitude to which the fluctuations within the complexes and MEVs are correlated [113].

## 3. Results

### 3.1. Epitope analyses

#### 3.1.1. Identification of potential virulent outer membrane peptides

A total of 49 potentially virulent MmpL proteins were identified. Following alignment, there were 465 peptides with varying lengths. There were 237 peptides that were determined to have an outer topology.

#### 3.1.2. Identification and analysis of the T-cell epitopes

There were 1127 CD8^+^ T-cell and 2040 CD4^+^ T-cell epitopes identified to be MHC I and II binders, respectively. Following antigenicity and immunogenicity analysis of the CD8^+^ T-cell epitopes, 464 and 321 epitopes were identified, respectively. There were 806 antigenic CD4^+^ T-cell epitopes, of which 259 were capable of potentially inducing IFN-γ and 128 were capable of potentially inducing IL-4. A total of 236 CD8^+^ and 84 CD4^+^ T-cell epitopes were determined to be non-allergenic and non-toxins. There were 36 conserved CD8^+^ T-cell epitopes, which overlapped with 46 CD4^+^ T-cell epitopes. Upon completion of the filtration of T-cell epitopes, there were 11 CD8^+^ and 13 CD4^+^ T-cell epitopes. There were 8 conserved CD8^+^ and 12 overlapping CD4^+^ T-cell epitopes (electronic supplementary material, table S4). The antigenicity scores of the CD8^+^ epitopes ranged from 3.89 (WFWWSPPPF) to 1.80 (RVRTRPTRT), and those of the CD4^+^ epitopes ranged from 2.21 (WFWWPMRVRTRPTRT) to 1.54 (FWWPMRVRTRPTRTP) (electronic supplementary material, table S4). The highest IC_50_ score for the CD8^+^ epitopes was 231.45 (RWFWWPMRV), and the lowest was 17.01 (WFWWPMRVR). The IC_50_ scores of the CD4^+^ epitopes ranged from 249.13 (LLGRWFWWPLRVRSR) to 20.00 (RWFWWPMRVRTRPTR). The identified CD8^+^ and CD4^+^ T-cell epitopes were traced back to 11 potentially virulent MmpL proteins, of which there was an overlap of proteins between the CD8^+^ and CD4^+^ epitopes (electronic supplementary material, table S6).

#### 3.1.3. Population coverage of the T-cell epitopes

**Figure 1.**
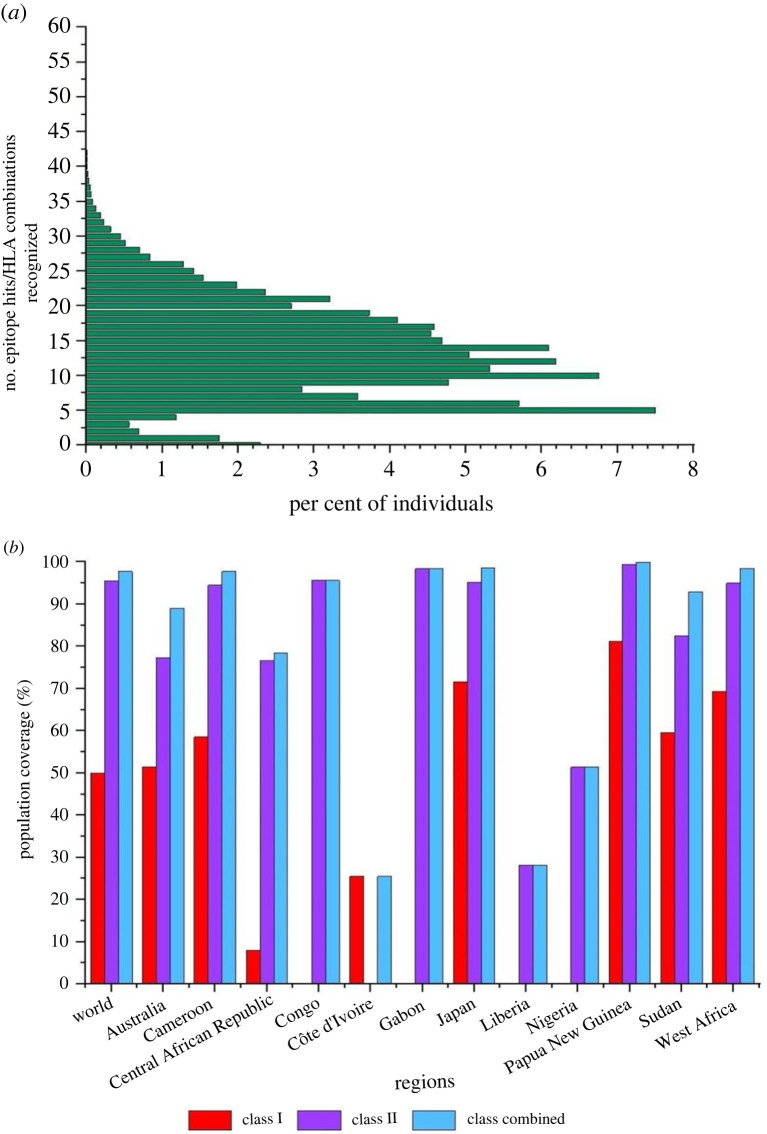
The population coverage of the CD8^+^ and CD4^+^ epitopes for the world and identified endemic regions. (*a*) The combined class HLA coverage for the world population. (*b*) The population coverage of individual and combined epitopes for the significant regions.

The highest number of epitope hits or HLA combinations recognized by the highest percentage of individuals was 5 (7.50%), and the lowest, excluding 0, were 40 to 42 (0.01%) (figure 1*a*). From 43 through to 60 epitope hits/HLA combinations, the number of individuals was 0 (figure 1*a*). The global class I, class II and class combined population coverages were 49.96%, 95.43% and 97.71%, respectively (figure 1*b*; electronic supplementary material, table S7). All the global estimates were above the estimated average (electronic supplementary material, table S7). Papua New Guinea had the highest class combined population coverage (99.90%), while Liberia had the lowest (28.23%) (figure 1*b*; electronic supplementary material, table S7).

#### 3.1.4. Identification and analysis of B-cell epitopes

A total of 334 B-cell epitopes were identified by ABCpred. These epitopes were then screened for antigenicity and reduced to 138 B-cell epitopes. There were 93 non-allergenic and non-toxic epitopes. Upon completion of conservancy analysis, 5 overlapping B-cell epitopes were identified (electronic supplementary material, table S8). The antigenicity scores of the epitopes ranged from 2.06 (WFWWPMRVRTRPTRTP) to 1.28 (MTPAIAALLGRWFWWP) (electronic supplementary material, table S8). The highest ABCpred score was 0.92 (MMPAIAALLGRWFWWP and MTPAIAALLGRWFWWP), and the lowest was 0.82 (LGRWFWWSPPPFLRAS). The B-cell epitopes were traced back and found to originate from 11 potential virulent proteins (electronic supplementary material, table S6). The virulence scores of the source proteins of the T- and B-cell epitopes ranged from 0.50 (WP_157759966.1) to 1.10 (WP_240273203.1) (electronic supplementary material, table S6).

#### 3.1.5. Molecular docking of the T-cell epitopes

**Figure 2.**
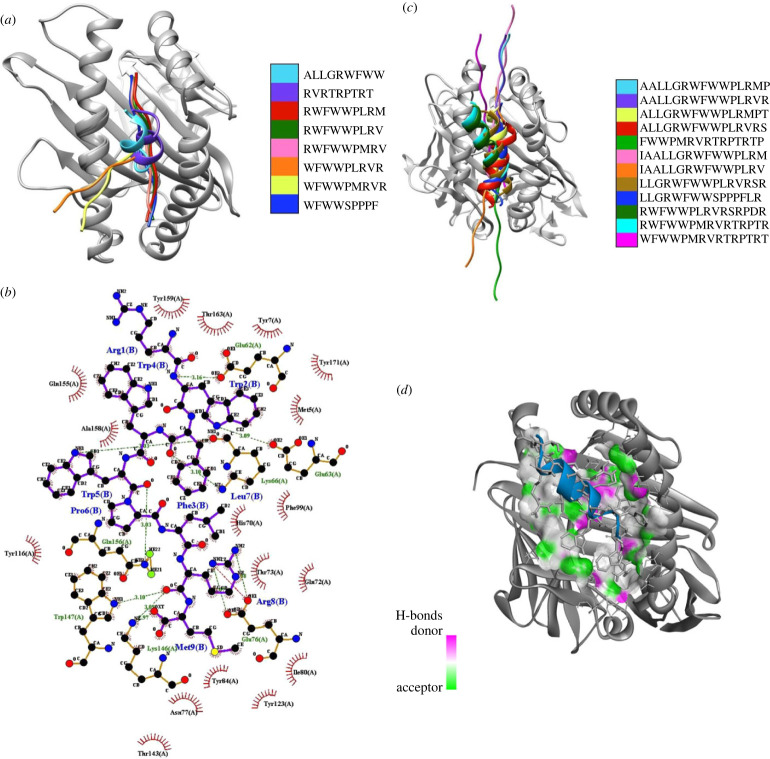
Superimposition of the most suitable epitopes and the binding interactions of the most promising epitopes. (*a*) The CD8^+^ epitopes are superimposed, with each colour representing a different model. The respective HLA allele structures are shown in grey. (*b*) The LigPlot depicting the interaction between the residues of the receptor and ligand (RWFWWPLRM). Hydrogen bonds are depicted in green and salt bridges in red. The residues involved in the hydrogen bonds are shown in green for the ligand and blue for the epitope. (*c*) The CD4^+^ epitopes are superimposed, with each colour representing a different model. The HLA allele structures are shown in grey. (*d*) The depiction of the RWFWWPMRVRTRPTR epitope with the HLA allele. The hydrogen bonds between the donor and acceptor are indicated by pink and green, respectively. White indicates neutral atoms.

The identified CD8^+^ T-cell epitopes were successfully bound to the most conserved HLA allele (figure 2*a*). The binding energies of the CD8^+^ epitopes to HLA-A*24:02 ranged from −187.81 kcal mol^−1^ (RWFWWPLRM) to −112.00 kcal mol^−1^ (WFWWPLRVR) (table 1). The residues involved in hydrogen bonds between the most promising epitope (RWFWWPLRM) and the HLA allele were Glu62, Glu63, Glu76, Gln156, Lys66, Lys146 and Trp147 (figure 2*b*). The CD4^+^ T-cell epitopes were also successfully bound to the most conserved HLA allele (figure 2*c*). The binding energies of the CD4^+^ epitopes to HLA-DRB1*15:01 ranged from −126.59 (ALLGRWFWWPLRMPT) to −179.55 (RWFWWPMRVRTRPTR) (table 1). The hydrogen bond interactions between RWFWWPMRVRTRPTR and the HLA allele were visualized, with the interactions between the donor and acceptor highlighted (figure 2*d*).

**Table 1.** The CD8^+^ and CD4^+^ epitopes, along with the best energy values. The CD8^+^ epitopes consist of 9 amino acids, and the CD4^+^ epitopes consist of 15 amino acids.

epitope	energy (kcal mol^−1^)
**CD8^+^ epitopes**	
ALLGRWFWW	−137.71
RVRTRPTRT	−113.68
RWFWWPLRM	−187.81
RWFWWPLRV	−171.98
RWFWWPMRV	−176.67
WFWWPLRVR	−112.00
WFWWPMRVR	−113.81
WFWWSPPPF	−147.22
**CD4^+^ epitopes**	
AALLGRWFWWPLRMP	−139.38
AALLGRWFWWPLRVR	−166.43
ALLGRWFWWPLRMPT	−126.59
ALLGRWFWWPLRVRS	−126.85
FWWPMRVRTRPTRTP	−158.31
IAALLGRWFWWPLRM	−138.91
IAALLGRWFWWPLRV	−145.22
LLGRWFWWPLRVRSR	−156.12
LLGRWFWWSPPPFLR	−167.18
RWFWWPLRVRSRPDR	−148.91
RWFWWPMRVRTRPTR	−179.55
WFWWPMRVRTRPTRT	−138.61

### 3.2. Multi-epitope vaccine sequence analyses

#### 3.2.1. Construction and structural analyses of the MEV candidate sequences

**Figure 3.**
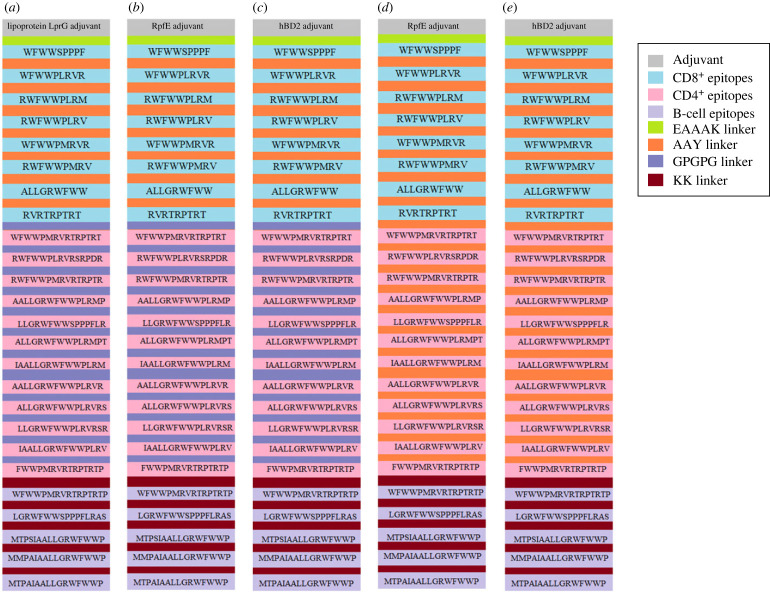
Schematic representation of the five multi-epitope vaccine constructs and a legend indicating the epitopes and linkers. (*a*) The schematic model of vaccine construct one, utilizing all the linkers. (*b*) The schematic model of vaccine construct two, utilizing all the linkers. (*c*) The schematic model of vaccine construct three, utilizing all the linkers. (*d*) The schematic model of vaccine construct four, utilizing the EAAAK, AAY and KK linkers. (*e*) The schematic model of vaccine construct five, utilizing the EAAAK, AAY and KK linkers.

**Figure 4.**
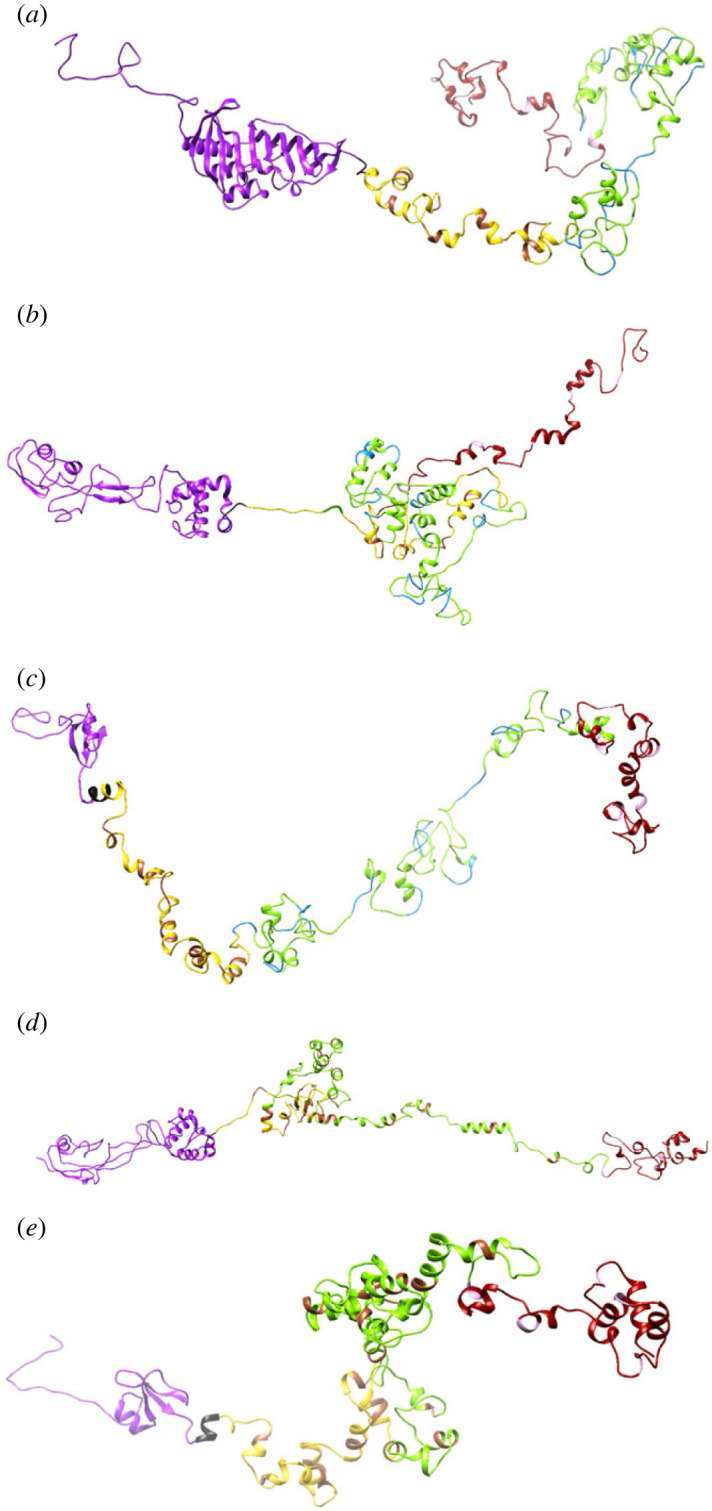
The refined three-dimensional models for the multi-epitope vaccines. The structures consist of the respective adjuvants (purple), EAAAK linker (black), CD8^+^ T-cell epitopes (yellow), AAY linkers (brown), CD4^+^ T-cell epitopes (green), GPGPG linkers (blue), KK linkers (pink) and the B-cell epitope (red). (*a*) The refined model of construct 1. (*b*) The refined construct of construct 2. (*c*) The refined structure of construct 3. (*d*) The refined model of construct 4. (*e*) The refined structure of construct 5.

Five vaccine sequences were constructed, as shown in figure 3. All the candidate sequences were found to be non-allergenic. The antigenicity scores of the candidates ranged from 1.42 (MEV3) to 1.17 (MEV1 and MEV4) (table 2). All the constructs consisted mainly of random coils, followed by extended strands and then alpha helices (table 2). MEV5 had the highest percentage of alpha helices (22.25%) and the lowest percentage of random coils (42.16%) (electronic supplementary material, figure S1). The sequences were refined (figure 4). The PROSA scores for the candidates were negative (table 2; electronic supplementary material, figure S2). The lowest score was −3.98 (MEV4), and the highest was −1.44 (MEV3) (table 2; electronic supplementary material, figure S2). The overall quality factors ranged from 64.60 (MEV4) to 74.16 (MEV5) (table 2; electronic supplementary material, figure S3). Ramachandran plots were generated for the refined candidate structures (electronic supplementary material, figure S4). MEV5 was the only construct with no residues in the disallowed regions and had the lowest percentage of residues in the generously allowed regions (table 2). MEV3 had the highest percentage of residues in the most favoured regions, while MEV2 had the lowest percentage of residues in this region and the highest percentage in the disallowed regions (table 2).

**Table 2.** The antigenic scores and structural analyses on the refined structures of the non-allergenic multi-epitope vaccine sequences.

vaccine candidate sequence number	antigenicity score	percentage of secondary structures			PROSA score	ERRAT overall quality factor	percentage of residues in the various regions of the Ramachandran plots			
		alpha helix (%)	extended strands (%)	random coils (%)			most favoured regions (%)	additional allowed regions (%)	generously allowed regions (%)	disallowed regions (%)
MEV1	1.17	12.95	34.79	52.26	−3.89	70.74	93.30	5.70	0.40	0.60
MEV2	1.20	7.63	33.96	58.41	−2.88	64.91	86.70	10.30	0.90	2.10
MEV3	1.42	6.05	40.73	53.23	−1.44	69.94	94.80	3.60	1.10	0.50
MEV4	1.17	20.06	29.77	50.16	−3.98	64.60	88.70	9.10	1.00	1.20
MEV5	1.39	22.25	35.59	42.16	−3.15	74.16	94.50	5.20	0.20	0.00

#### 3.2.2. Physico-chemical analysis of the vaccine candidate sequences

The vaccine sequences consisted of varying numbers of amino acids (table 3). All the vaccine sequences had theoretical pI values ≥7, II values ≥40 and negative GRAVY values, indicating their basic, unstable, and hydrophilic natures (table 3). The molecular weight of the sequences ranged from 58.74 kDa (MEV5) to 76.53 kDa (MEV1) (table 3). The aliphatic index values for the constructs indicate varying levels of thermostability, with MEV5 having the greatest value (69.84) and MEV2 the lowest (59.27) (table 3). The N-terminal for all the sequences was considered to be methionine (M). The half-life estimated in mammalian reticulocytes (*in vitro*), yeast (*in vivo*) and *E. coli* (*in vivo*) were 30 h, greater than 20 h and greater than 10 h, respectively. Gibbs–Helmholtz curves were generated based on the five vaccine candidate structures (electronic supplementary material, figure S5). MEV4 had the highest melting temperature (65.00°C), while MEV3 had the lowest melting temperature (52.70°C) (electronic supplementary material, table S9). Only MEV2 had negative values for ΔH_m_, ΔC_p_ and *Δ*G_r_, as indicated by the difference in the curve (electronic supplementary material, table S9, figure S5B).

**Table 3.** The physico-chemical properties of the five vaccine candidate sequences. pI = isoelectric point, II = instability index, GRAVY = grand average of hydropathicity.

vaccine candidate	number of amino acids	molecular weight (kDa)	theoretic pI	II	aliphatic index	GRAVY
MEV1	664	76.53	12.02	42.45	68.83	−0.27
MEV2	642	73.59	11.82	55.18	59.27	−0.33
MEV3	496	59.46	12.39	51.42	61.43	−0.22
MEV4	618	72.87	11.48	60.12	65.45	−0.22
MEV5	472	58.74	12.05	57.71	69.64	−0.06

#### 3.2.3. Codon adaptation, vaccine optimization and cloning

**Figure 5.**
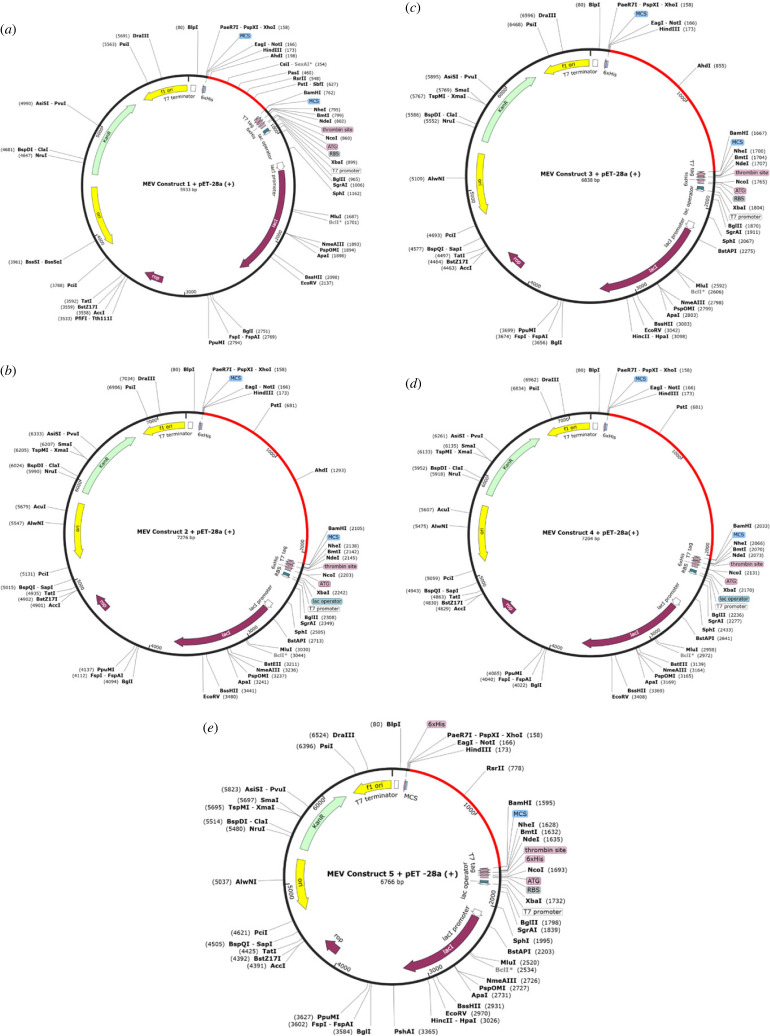
The *in silico* cloning map of the pET-28a(+) plasmid, with the optimized DNA sequence shown in red. (*a*) The cloning map of the optimized DNA sequence of the first MEV construct. The sequence is located between HindIII (173) and BamHI (762). (*b*) The cloning map of the optimized DNA sequence of the second MEV construct. The sequence is located between HindIII (173) and BamHI (2105). (*c*) The cloning map of the optimized DNA sequence of the third MEV construct. The sequence is located between HindIII (173) and BamHI (1667). (*d*) The cloning map of the optimized DNA sequence of the fourth MEV construct. The sequence is located between HindIII (173) and BamHI (2033). (*e*) The cloning map of the optimized DNA sequence of the fifth MEV construct. The sequence is located between HindIII (173) and BamHI (1595).

The codon adaptation index (CAI) value for all the vaccine candidate sequences was 1. The GC content was 60.89 (MEV1), 63.71 (MEV2), 62.77 (MEV3), 61.65 (MEV4) and 60.03 (MEV5). The sequences were cloned *in silico* into the pET-28a(+) plasmid (figure 5). The length of the maps varied between 5933 bp (MEV1), 7276 bp (MEV2), 6838 bp (MEV3), 7204 bp (MEV4), 6766 bp (MEV5) (figure 5).

#### 3.2.4. Binding interaction of constructed multi-epitope to the TLRs and structural analysis of the TLR–MEV complex

**Figure 6.**
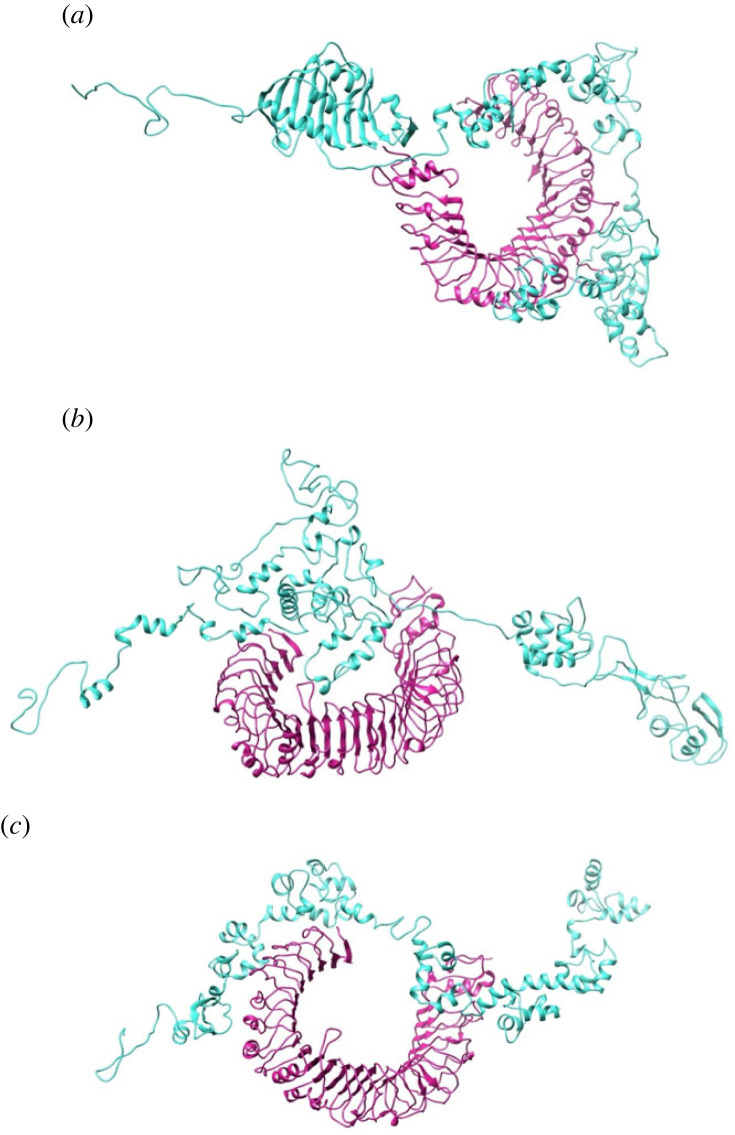
The crystalline structures of the MEV–TLR complexes. The multi-epitope chain is shown in red and the respective TLR is shown in blue. (*a*) The first MEV–TLR2 complex. (*b*) The second MEV–TLR4 complex. (*c*) The third MEV–TLR4 complex.

**Figure 7.**
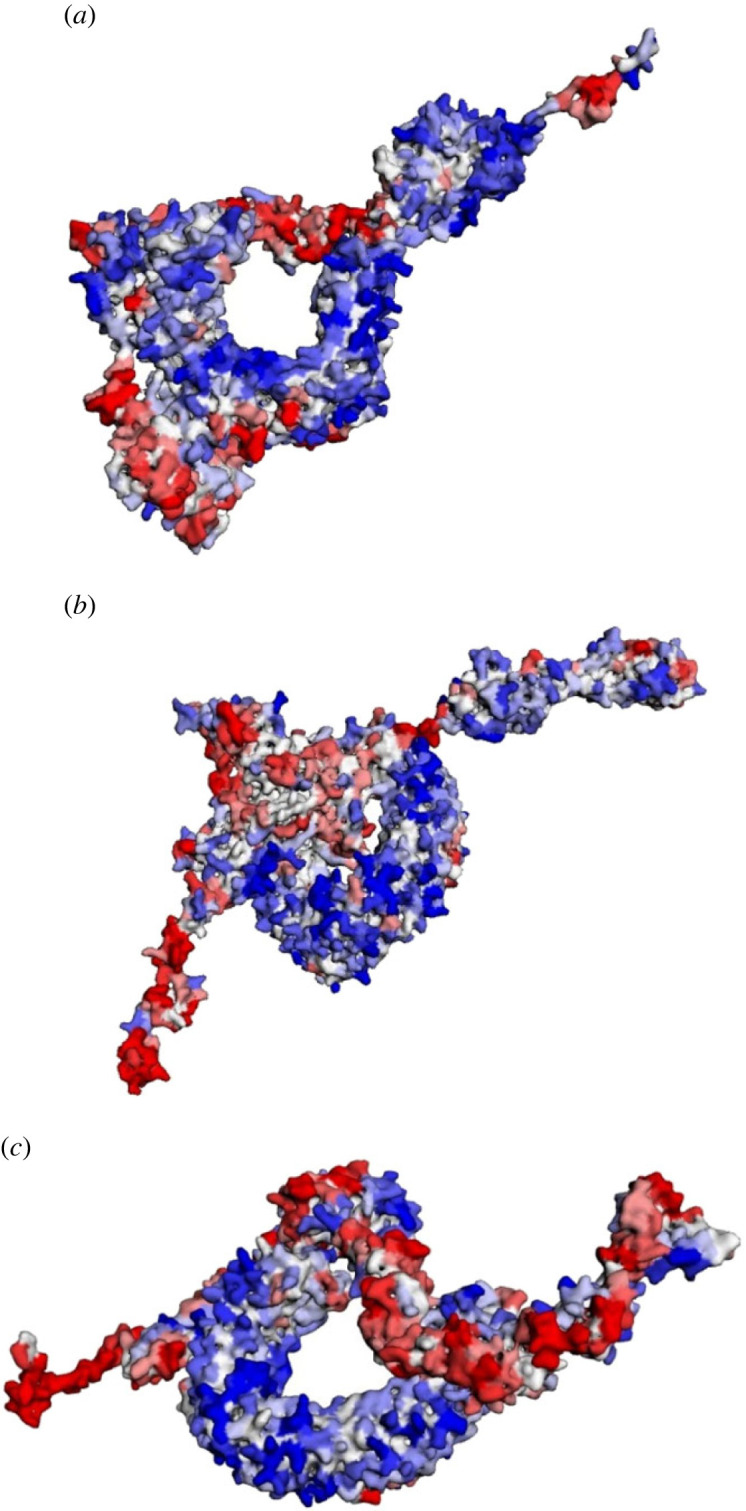
The solubility and aggregation propensity of the MEV–TLR complexes. The graphical representation model shows the soluble residues in red, the aggregation-prone residues in blue, and residues with no predicted influence shown in white. (*a*) The model based on the first MEV–TLR2 complex. (*b*) The model based on the second MEV–TLR4 complex. (*c*) The model based on the third MEV–TLR4 complex.

Only three vaccine sequences (MEV1, MEV2 and MEV5) were successfully docked to their respective TLR, resulting in complex 1, complex 2 and complex 3, respectively (figure 6). The best binding energies of the abovementioned complexes were −266.68, −382.36 and −368.67, respectively. Complex 1 consisted of 3 salt bridges, 6 hydrogen bonds and 119 non-bonded contacts (electronic supplementary material, figure S6A). Complex 2 contained 3 salt bridges, 3 hydrogen bonds and 142 non-bonded contacts (electronic supplementary material, figure S6B). Complex 3 had the highest numbers of bonds with 5 salt bridges, 9 hydrogen bonds and 131 non-bonded contacts (electronic supplementary material, figure S6C). The contact maps generated based on the complexes indicate varying degrees of contact between the residues (electronic supplementary material, figure S7). Complex 1 had a minimal score of −3.93, a maximal score of 3.54, an average score of −0.54 and a total score of −658.74 (figure 7*a*). The minimal, maximal, average, and total scores of complex 2 were −3.59, 3.25, −0.47, and −585.18, respectively (figure 7*b*). Complex 3 had a minimal score of −3.70, a maximal score of 5.35, an average score of −0.31, and a total score of −331.19 (figure 7*c*). Complex 1 had the highest number of soluble residues, while complex 3 had the lowest (table 4). Complex 3 had the highest aggregation-prone residues, while complex 1 had the lowest (table 4).

**Table 4.** Aggregation analysis regarding the three complexes and individual chains A and B. AP = aggregation prone, NI = negligible influence.

complex	complex			chain A			chain B		
	AP	NI	soluble	AP	NI	soluble	AP	NI	soluble
1	210	344	660	34	198	318	176	146	342
2	220	397	626	36	239	326	184	158	300
3	263	322	488	29	239	333	234	83	155

#### 3.2.5. Immune simulations

**Figure 8.**
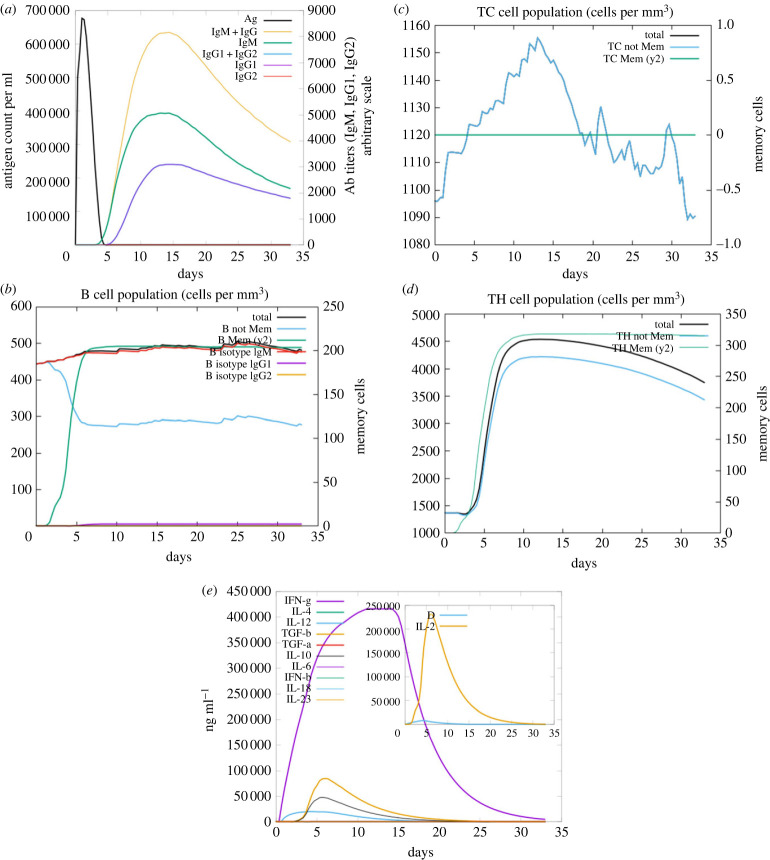
The immune simulation results from C-IMMSIM of MEV construct one. (*a*) The induced antigen and immunoglobin responses. (*b*) The B-cell population: total count, memory cells, and sub-divided in isotypes IgM, IgG1 and IgG2. (*c*) The CD8^+^ cytotoxic lymphocyte count. (*d*) The CD4^+^ T-helper lymphocyte count. (*e*) The induced cytokine response. The inset plot shows the danger signal together with IL-2.

**Figure 9.**
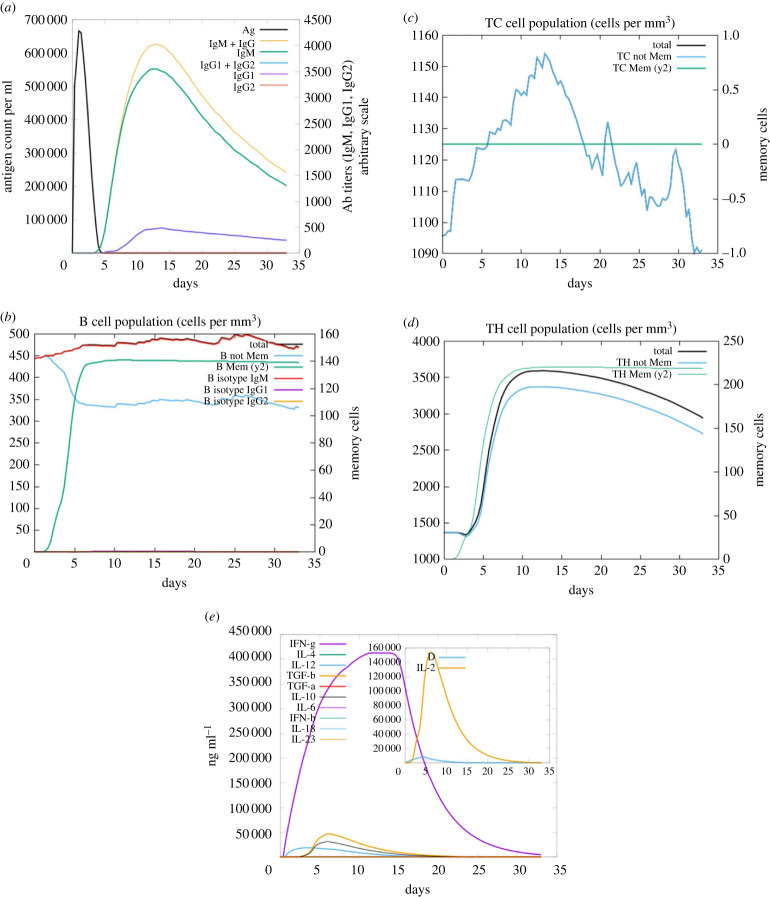
The immune simulation results from C-IMMSIM of MEV construct five. (*a*) The induced antigen and immunoglobin responses. (*b*) The B-cell population: total count, memory cells, and sub-divided in isotypes IgM, IgG1 and IgG2. (*c*) The CD8^+^ cytotoxic lymphocyte count. (*d*) The CD4^+^ T-helper lymphocyte count. (*e*) The induced cytokine response. The inset plot displays the danger signal coupled with IL-2.

Immune simulations were carried out for MEV1, MEV2, and MEV5 (figures 8 and 9; electronic supplementary material, figure S8). All the vaccine sequences induced immunoglobin and antibody responses (figures 8*a* and 9*a*; electronic supplementary material, figure S8A). However, only MEV2 induced IgG1 + IgG2 and IgG2 and had the highest levels of IgM + IgG and IgM (electronic supplementary material, figure S8A). For all vaccine candidates, the antigen response increased and consequentially decreased within the 0 to 5-day period (figures 8*a* and 9*a*; electronic supplementary material, figure S8A). All vaccine sequences induced the production of memory, non-memory, and B isotype IgM B-cells (figures 8*b* and 9*b*; electronic supplementary material, figure S8B). MEV2 generated the lowest number of B isotype IgM and memory B-cells (figure 9*b*). The production of B isotype IgG1 and IgG2 was negligible (figures 8*b* and 9*b*; electronic supplementary material, figure S8B). The population of memory CD8^+^ T-cells remained constant throughout the simulation for all the sequences (figures 8*c* and 9*c*; electronic supplementary material, figure S8C). The induction of non-memory CD8^+^ T-cells fluctuated throughout the simulation, and the values were similar (figures 8*c* and 9*c*; electronic supplementary material, figure S8C). All the sequences induced the production of memory and non-memory CD4^+^ T-cells; however, MEV5 induced the lowest number of both types of cells (figures 8*d* and 9*d*; electronic supplementary material, figure S8D). The vaccine sequences induced the production of IFN-γ, transforming growth factor beta (TGF-β), IL-2, IL-10, and IL-18 (figures 8*e* and 9*e*; electronic supplementary material, figure S8E). The levels of IFN-γ and IL-18 induced were similar for all the sequences; however, MEV5 induced the lowest amount of IL-10 and TGF-β (figures 8*e* and 9*e*; electronic supplementary material, figure S8E).

### 3.3. Molecular dynamics simulations

**Figure 10.**
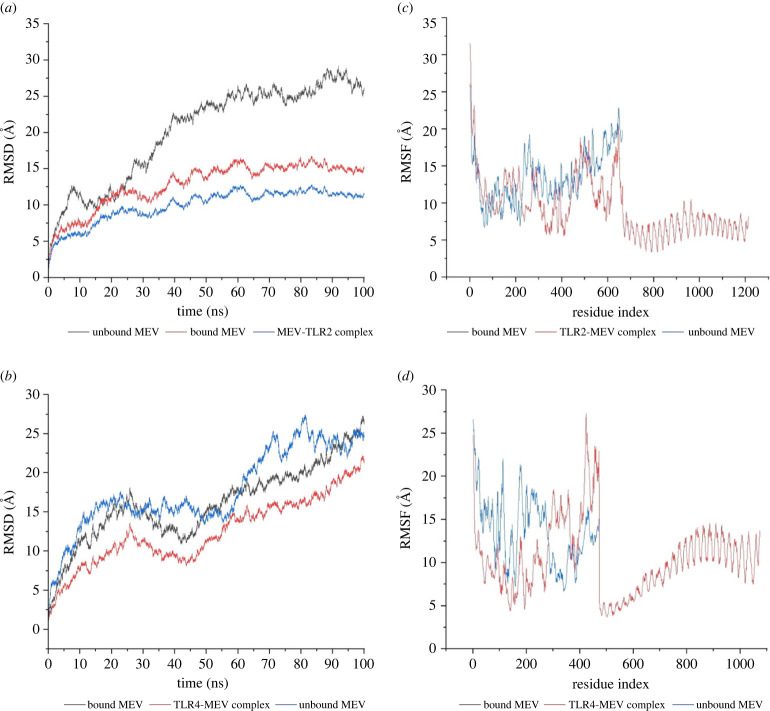
The molecular dynamics simulations of the MEV–TLR docked complexes, MEV bound to TLR and the unbound MEV. (*a*) The RMSD plot was generated based on complex one. The bound MEV is shown in red, the unbound MEV in grey and the TLR2–MEV complex in blue. (*b*) The RMSD plot was generated based on complex three. The bound MEV is shown in grey, the unbound MEV in blue and the TLR4–MEV complex in red. (*c*) The RMSF plot was generated based on complex one. The bound MEV is shown in grey, the unbound MEV in blue and the TLR2–MEV complex in red. (*d*) The RMSF plot was generated based on complex three. The bound MEV is shown in grey, the unbound MEV in blue and the TLR4–MEV complex in red.

The RMSD values of complex one and the bound MEV were lower than that of the unbound MEV (figure 10*a*). However, there was an overlap of the RMSD values of the bound and unbound for the third complex (figure 10*b*). The fluctuations of the backbone of the atoms of the bound MEV1 ranged from 0.55 Å to 16.09 Å, while the fluctuations of the backbone of the atoms of the unbound MEV1 ranged from 0.55 Å to 27.75 Å (figure 10*a*). The highest value for the fluctuations of the backbone of the atoms for the bound MEV5 was 0.49 Å, and the lowest was 22.74 Å (figure 10*b*). The fluctuations of the backbone of the atoms of the unbound MEV5 ranged from 0.52 Å to 24 Å (23.9954) (figure 10*b*). These values indicate better stability of the bound MEVs. Residues within the third complex displayed lower fluctuations than the first complex (figure 10*c*,*d*). There was also an overlap between complex one and the unbound MEV1 (figure 10*c*).

### 3.4. Post-molecular dynamics simulations analysis

**Figure 11.**
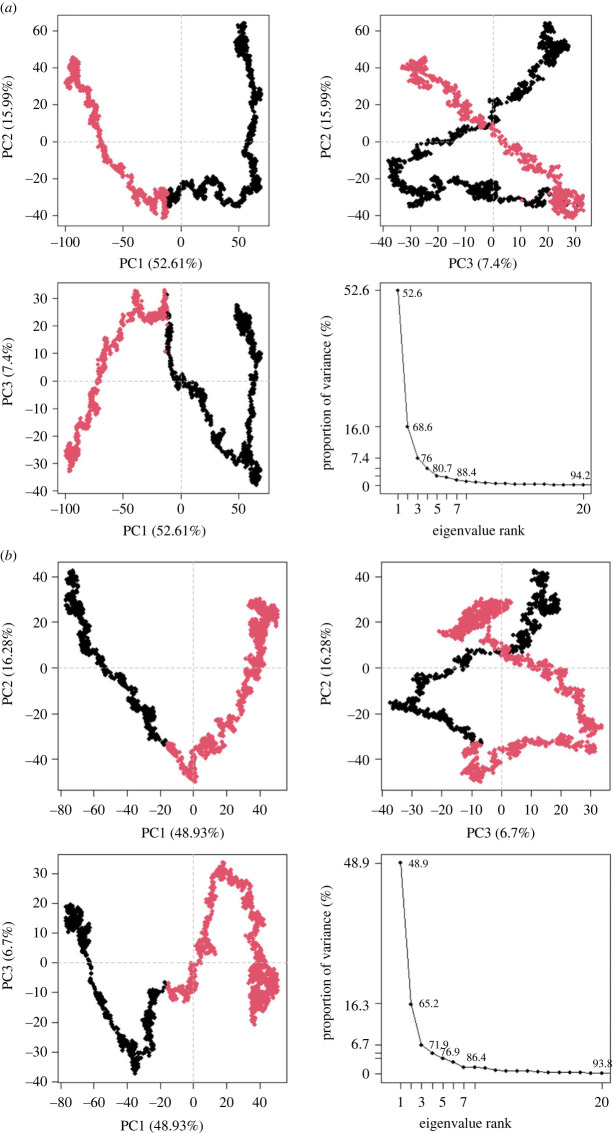
The PCA plots generated for the MEV constructs in eigenvalue rank; PC2 versus PC1, PC2 versus PC3, PC3 versus PC1. The colours are based on order of time and the cumulative variability at each data point. (*a*) The PCA plot based on the first MEV construct. (*b*) The PCA plot based on the third MEV construct.

**Figure 12.**
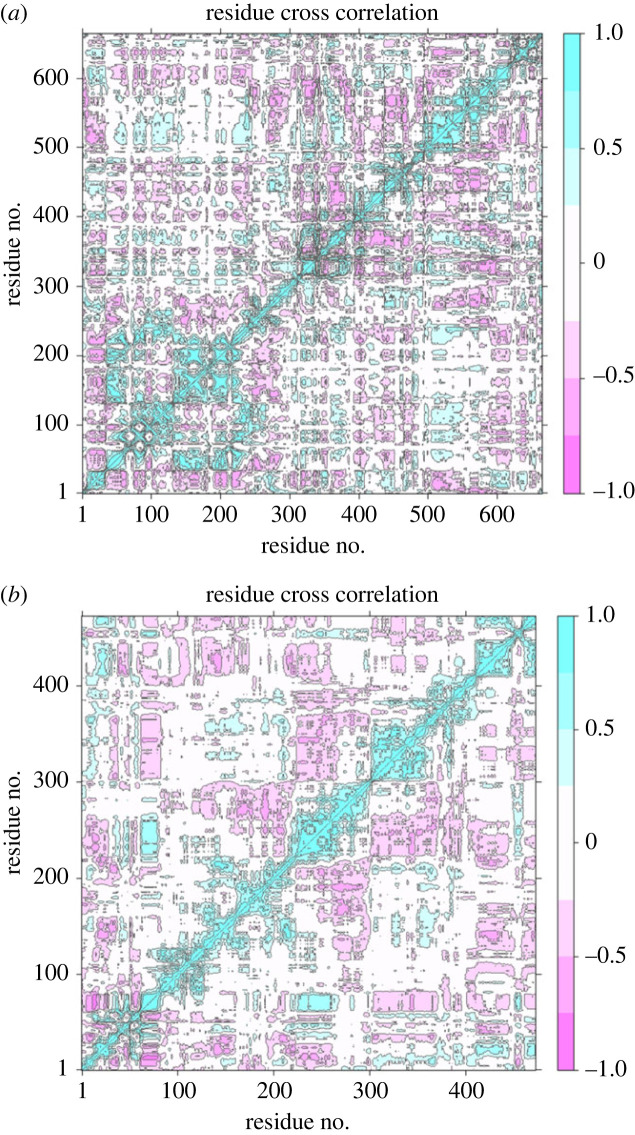
The dynamical cross-correlation map generated based on the MEV constructs. The blue regions indicate the residues moving in a singular direction, while the pink regions indicate that the residues moved in opposite directions. (*a*) The DCCM generated from MEV1. (*b*) The DCCM generated from MEV2.

MM/GBSA was used to estimate the binding affinities of the vaccine sequences to their respective TLRs within the complex (table 5). The endpoint energies for complexes indicate good interactions between the TLRs and vaccine sequences. The first complex displayed better binding interactions than the third complex (table 5). Both complexes had negative van der Waals energies, which is favourable (table 5). The electrostatic, gas-phase and solvation free energy contributions are shown to be significant. Principal cross-correlation captured 94.20% of the variance of the atom positional fluctuations of the first vaccine sequence during MDS (figure 11*a*). PC1 to PC3 contributed 76% of the total proportion of the variance shown in the eigenvalue plot (figure 11*a*). The greatest contribution was by PC1 (52.61%), followed by PC2 (15.99%) and then PC3 (7.40%) (figure 11*a*). This differed from the principal cross-correlation captured for complex one, which was 92.10 (electronic supplementary material, figure S9A). The total contributions of PC1 to PC3 were estimated to be 72.68% (electronic supplementary material, figure S9A). PC1 contributed 51.77%, PC2 contributed 16.09%, and PC3 contributed 4.82% (electronic supplementary material, figure S9A). The fifth vaccine sequence had a lower proportion of variance than the first, i.e. 93.80% (figure 11*b*). The three PCs were accountable for 71.91% (figure 11*b*). PC1 contributed the highest variability, followed by PC2 and PC3, with proportions of 48.93%, 16.28% and 6.70%, respectively (figure 11*b*). The variance of the third complex was lower, with the 20 PCs capturing 89% of the variance (electronic supplementary material, figure S9B). PC1 to PC3 contributed 64.47% of the variance (electronic supplementary material, figure S9B). Similar to the other plots, PC1 contributed the highest (48.08%), then PC2 (12.98%), and PC3 (6.41%). Residues within the complexes and vaccine constructs were observed to move in similar directions near the diagonal, indicating the inter-correlation of residues (figure 12; electronic supplementary material, figure S10). Various residual-wise loading fluctuations were observed for the complexes and vaccine constructs (electronic supplementary material, figure S11).

**Table 5.** The energy composition profile (kcal mol^−1^) based on the MM/GBSA analysis of the two vaccine complexes. Δ*E_VDW_* = the van der Waals contribution from MM. Δ*E_ELE_* = the electrostatic energy calculated by the molecular mechanics (MM) force field. Δ*G*_gas_ = the gas-phase energy contribution. Δ*G*_solv_ = the solvation free energy contribution. Δ*G*_bind_ = the endpoint binding energy of the interaction of the complex.

energy component	MEV–TLR2 complex	MEV–TLR4 complex
Δ*E_VDW_*	−269.02 ± 0.76	−216.32 ± 0.96
Δ*E_ELE_*	−4003.63 ± 5.25	−10672.74 ± 15.11
Δ*G*_gas_	−4272.65 ± 5.87	−10889.07 ± 16.00
Δ*G_s_*_olv_	4110.57 ± 5.38	10747.06 ± 15.23
Δ*G*_bind_	−162.08 ± 0.60	−142.00 ± 0.86

## 4. Discussion

BU is a disease that burdens individuals and communities physically, economically, and socially. The progression of technology has given rise to new methods that can supplement existing vaccine methodologies while lowering production costs and time. One such method, i.e. immunoinformatics, was used in this study to generate multiple *in silico* vaccine constructs, which may be capable of inducing protective immune responses against *M. ulcerans* infection. The recognition of a vaccine by the immune system of the host is essential for its effectiveness [116]. The host immune system is comprised innate and adaptive immunity. T-cells and B-cells play crucial parts in removing infection in the host through the adaptive immune response [117]. The role of CD8^+^ or cytotoxic T-cells is to recognize and destroy infected cells, while CD4^+^ or helper T-cells are involved in the differentiation of B-cells and cytokine induction [1]. This constitutes the cell-mediated response; the humoral antibody response consists of B-cells which are responsible for the generation of antigen-specific antibodies [1,117]. These antibodies are responsible for the neutralization of infection prior to its spread [118].

Upon infection with *M. ulcerans,* immune suppression may occur due to the toxin mycolactone [119]. The immune response of the host has been found to correspond to the stage of the disease, with a predominantly TH2 response during the initial progression of the disease and a TH1 response during the later or healing stages of the disease [120]. This TH2 response has been coupled with increased IL-10, while the TH1 response corresponds with the secretion of high levels of IFN-γ [120]. IFN-γ is critically involved in protection against mycobacteria, with a lack of IFN-γ in patients possibly contributing to the persistence of *M. ulcerans*, the painless characterized by the disease and the failure of the disease to respond to treatment [119]. Higher levels of IFN-γ were observed in patients in ulcerative rather than pre-ulcerative phases and in late BU or healed patients when compared to controls [120]. This differed from the findings of decreased IFN-γ production in patients with ulcerative lesions compared to nodules [121]. This was the opposite for the IL-10 cytokine, with increased levels found in patients with ulcerative lesions than with nodules [121]. The generation of conserved CD8^+^ T-cell epitopes with overlapping CD4^+^ epitopes capable of inducing IFN-γ is attractive.

Antigenicity is another desirable property, as it is the ability of an antigen to interact specifically with the binding site of an antibody molecule [122]. The epitopes identified in this study have varying antigenicity scores. The non-allergenic and non-toxic nature of the epitopes, and corresponding vaccine constructs, indicate a lower probability of the induction of adverse effects. The epitopes in this study share several properties with previously identified epitopes [37–39]. This includes antigenicity, non-allergenicity, non-toxicity, cytokine-inducing capabilities, and some degree of population coverage. However, none of the final epitopes generated in this study were shared with prior studies [37,38]. This may be due to the inclusion of other strains of *M. ulcerans* and the use of a different protein, i.e. MmpL. While our previous study used the same strains of *M. ulcerans* [39]*,* there were a larger number of epitopes which displayed the abovementioned desirable properties identified in this study. The resulting vaccine candidates displayed similar structural properties; however, overlap of the RMSD values was only observed in this study. This may be linked to the greater size of these vaccine candidates or the quality of the structure.

Different species of mycobacteria are observed to have varying numbers of MmpL proteins, with the H37Rv stain of *M. tuberculosis* having 13, *M. leprae* with 5, *M. smegmatis* with 16, and *M. abscessus* with approximately 31 putative transporters [41,123]. However, 6 MmpL transporters (MmpL 3/4/7/10/11 and 13a/13b) are thought to form the core set of this protein, as there is a high degree of syntenic conservation in both slow-growing and rapidly growing mycobacteria [40,41]. This was observed by examining the MmpL proteins of *M. tuberculosis, M. leprae, M. avium, M. marinum, M. smegmatis* and *M. abscessus* [40,41]. Little is known regarding their structural organization, topological architecture and mechanism of action in mycobacteria [123]. MmpL proteins are found to indirectly contribute towards virulence through their effect on bacterial survival [41]. These proteins are also associated with drug resistance through their impact on the efflux of antibiotics from the periplasm [124]. The use of epitopes derived from these proteins in the vaccine candidates is promising.

Another promising characteristic is the high population coverage of these epitopes in most endemic regions. WHO has reported incidences of BU in 33 countries, including Australia, Benin, Cameroon, Central African Republic, Congo, Côte d'Ivoire, Democratic Republic of the Congo, Gabon, Ghana, Guinea, Japan, Liberia, Nigeria, Papua New Guinea, Togo, French Guiana and South Sudan [14,58]. The regions identified as endemic were selected for population coverage analysis; however, no population coverage was generated for Ghana. This may be due to the lack of the use of HLA alleles within this study, as the Accre Asutuare Akan population did not show any alleles under the gold or gold and silver population standards [46]. The Ga-Adangbe population of Ghana was missed when identifying the most frequent HLA alleles in the population; however, this can be addressed in later studies. Togo and Benin were not featured on the population coverage webserver [57]. The inclusion of West Africa was done to encompass Benin and Ghana. The class combined population of West Africa (98.45%) was high. When compared to the study by Nain *et al*. [38], the global class I population coverage (49.96%) and the class combined population coverage (97.71%) were lower, but the class II population coverage was higher (95.43%). The individual and global population coverages indicate the potential of some degree of effectiveness in the endemic areas.

The epitopes that displayed the abovementioned desirable properties were combined, together with linkers and adjuvants, to form several vaccine candidate sequences. The addition of the adjuvants was done to increase the stimulation of the immune response, thereby increasing the immunogenicity of the vaccine construct [117,125]. TLR agonists are shown to be promising adjuvants due to their ability to induce antigen-presenting cells (APCs) [126]. The lipoprotein LprG was one of the adjuvants used, as it is a TLR2 agonist. Brightbill *et al*. [127] found that the use of microbial lipoproteins to activate TLRs may lead to the induction of innate defence mechanisms against pathogens. LprG has been observed to aid the immune system in recognizing *M. tuberculosis* [72]. RpfE was also used as an adjuvant due to its role as a TLR4 agonist. RpfE from *M. tuberculosis* is involved in the modulation of dendritic cell (DC) function, which facilitates the differentiation of CD4^+^ T-cells to Th1 or Th17 [126]. hBD2 was chosen as an adjuvant due to its antibacterial activity [128]. Defensins defend against pathogenic infection by linking both the adaptive and innate immune systems through T-cells and DCs [128]. Linkers serve to separate epitopes and facilitate folding to achieve epitope display [129]. The EAAAK linker is defined as a rigid linker and plays a role in protein stability by maintaining a distance between domains [130]. It is advised that it should be used after an adjuvant [130]. The AAY linkers are involved in the formation of natural epitopes while also limiting junctional epitope formation [131]. This was also observed with the GPGPG linker, which also aided in increasing the specificity of the immune response [132]. The KK linker was used to preserve the independent immunological activity of the epitopes [133]. Linkers have an impact on the hydrophobicity, secondary structures, sensitivity to protease, and the interactions within the regions of the vaccine constructs [134]. Some of these characteristics were further analysed.

The vaccine constructs displayed several physico-chemical properties. Proteins with molecular weights ≤110 kDa are desirable in vaccine development due to rapid purification [135,136]. All vaccine candidate sequences were below 110 kDa. It is found that the higher the aliphatic index, the more thermostable the protein is [137]. The varying levels of thermostability of the constructs are positive, as it was found that thermostable vaccines would not require cold temperature storage, thereby potentially decreasing medical costs and productivity losses while increasing the reach of the vaccine [138]. This, coupled with the high melting temperatures, is advantageous. The negative GRAVY values, indicative of hydrophilicity, indicate strong interaction with water molecules [139]. This allows for easy dispersion and mobility of the proteins [140]. All the vaccine candidate sequences were found to be unstable as per the II; however, this value is based on the dipeptide composition of the sequence and does not factor in different environmental conditions nor the secondary and tertiary structures of the protein [88,141]. It was found that *in vitro* conditions also impact the stability of a protein [141]. Stability of the vaccine candidates may be modulated by ensuring optimal environmental conditions [142]. The estimated half-life of the candidates in different organisms is positive, as it may ensure that the constructs will remain viable long enough to induce an effective immune response. However, this would need to be experimentally validated.

The CAI is used to measure codon usage bias, with it correlating positively to gene expression [143]. The high CAI values for each vaccine candidate indicate that they may adapt well to *E. coli* (strain K12) [143]. The GC content was within the range of 30–70%, indicating a high potential for reproducibility and good protein expression [144]. The simulated immune response of the vaccine candidates displayed the production of T-cells, B-cells, cytokines and immunoglobins. Several studies involving the immunization of *M. ulcerans-*specific candidates in animal models have observed the induction of specific immunoglobins, such as IgG, IgG1, IgG2a and IgG2b [25,27–29,32,34,36]. The release of immunoglobins has been observed in patients, healthy family members and the sera of patients with TB from non-endemic areas [145]. High levels of IgG responses were noted in all three groups, while IgM antibody responses were more distinct in patients [145]. IgG production in FVB/N mice has been observed during the healing stage of *M. ulcerans* infection [146]. It was hypothesized that this immunoglobin might contribute towards the control of *M. ulcerans* infection in this model [146]. The production of IgM, IgG1 and IgM and IgG are promising. However, only one dose of the vaccine candidates was run, with vaccines generally requiring more than a single dose before full protection is achieved.

The secondary structures of proteins impact protein folding and provide information regarding the functions and interactions of the protein [147]. The large proportion of random coils in the vaccine candidates indicates the high flexibility of the proteins [148]. The presence of alpha-helices is advantageous, as it indicates the maximum possible number of hydrogen bonds, indicative of stability [149]. Extended strands are common structural occurrences in proteins, and isolated extended strands often share characteristics of loops and *β*-sheets [150]. These secondary structures each affect the overall protein structure. The protein structures showed marked improvement following refinement, indicated by the Z-score and the Ramachandran plots. The negative Z-score indicates that the model quality of the proteins is good [84]. This was further emphasized with most residues lying within the most favoured regions of the Ramachandran plots. However, overall quality factors of 95% or higher are considered to show good high-resolution structures [151]. The lower quality factors of the vaccine candidates will need to be addressed in further studies.

TLRs are crucial in recognizing pathogenic signals and activating innate and adaptive immune responses [152]. Through the interaction of the TLR and vaccine antigens, Th1 cytokines may be produced [116,127]. TLR2 and TLR4 are expressed on the cell surface and primarily recognize lipids, lipoproteins and proteins that form the microbial membrane [116,153]. TLR2 is observed to play a role in the recognition and induction of the host immune response to mycobacteria [154,155]. This is done via the activation of the MyD88, IRAK4, TRAF6, TAK1, and NF-kB dependent and NF-κB independent signalling pathways [152,153]. TLR4 has been observed to induce host responses against *M. tuberculosis;* this includes the induction of apoptosis and anti-inflammatory responses in macrophages [152,156].

The bonds within the TLR–vaccine complexes consisted mainly of hydrogen bonds and salt bridges. Both of these types of interactions are involved in protein binding [157]. The presence of hydrogen bonds within proteins contributes to protein stability [158]. However, the contribution of each hydrogen bond is dependent on its distance and geometry [158]. Salt bridges are also found to contribute towards protein stabilization [159]. They are found to play a role in the folding of globular proteins, and may contribute towards membrane protein stability, conformational specificity, and the positioning of critical functional groups [160,161]. Another key characteristic within the complexes is the smaller proportion of aggregation-prone residues, as aggregation may lead to the reduction of production yield and final product activities and may trigger unpredictable immune responses in patients [162,163].

MDS was used to further analyse the interactions between the proteins and ligands and the conformational modification of the proteins at the atomic level [164]. The RMSD and RMSF values indicate the stability of the two complexes at the end of the simulation. However, as MDS often emulates the absolute atomistic level molecular behaviour in near-cytosolic conditions through the use of the relevant force-fields and water model, further physiological validation through *in vitro* and *ex vivo* studies is required [165,166]. MMGBSA estimates the free energy of the binding of small ligands to biological macromolecules [167]. In this study, it was used to estimate the free energy of the binding of the vaccine constructs and TLRs. The energy estimates showed significant contributions from the van der Waals forces, electrostatic energy, and gas-phase energy. Van der Waals interactions often correlate to the number of atoms, with lower van der Waals energies correlating to larger peptides [168]. These interactions are an important factor for binding affinity [168]. Hydrogen bonds and salt bridges are classified mainly as electrostatic interactions [158,160]. This may have contributed towards the significant electrostatic energy contributions.

BU remains a burden on not only affected individuals but communities as a whole. Early diagnosis and treatment prevent the development of severe symptoms. The prevention or control of the infection may also be beneficial. However, control and prevention methods remain lacking. The epitopes, and by extension, the vaccine constructs and complexes generated in this study displayed several appropriate characteristics. These characteristics suggest the ability of these constructs to generate a potentially protective immune response against *M. ulcerans* infection. This study serves to further the scope of existing screened epitopes. However, further experimental validation is required as this work was only done computationally.

## Data Availability

The datasets can be found at National Center for Biotechnology Information (NCBI) with the following accession numbers: GCA_022374915.1, https://www.ncbi.nlm.nih.gov/assembly/GCA_022374915.1; GCA_020150655.1, https://www.ncbi.nlm.nih.gov/assembly/GCA_020150655.1; GCA_020616615.1, https://www.ncbi.nlm.nih.gov/assembly/GCA_020616615.1; GCA_900638745.1, https://www.ncbi.nlm.nih.gov/assembly/GCA_900638745.1; GCA_001870585.1, https://www.ncbi.nlm.nih.gov/assembly/GCA_001870585.1; GCA_901411635.1, https://www.ncbi.nlm.nih.gov/assembly/GCA_901411635.1; GCA_902506705.1, https://www.ncbi.nlm.nih.gov/assembly/GCA_902506705.1; GCA_900683785.1, https://www.ncbi.nlm.nih.gov/assembly/GCA_900683785.1; GCA_000013925.2, https://www.ncbi.nlm.nih.gov/assembly/GCA_000013925.2; GCA_000524035.1, https://www.ncbi.nlm.nih.gov/assembly/GCA_000524035.1; GCA_002355775.1, https://www.ncbi.nlm.nih.gov/assembly/GCA_002355775.1; GCA_002356495.1, https://www.ncbi.nlm.nih.gov/assembly/GCA_002356495. Supplementary material is available online [169].
